# Red‐to‐Near‐Infrared Fluorescence Resonance Energy Transfer Reporters for Dual Imaging Applications

**DOI:** 10.1002/smtd.70828

**Published:** 2026-07-12

**Authors:** Van Thi‐Hong Tran, Frans Ek, Hasan Yassine, Rajalaxmi Behera, Vesa P. Hytönen, James R. W. Conway

**Affiliations:** ^1^ Department of Biochemistry and Developmental Biology Faculty of Medicine University of Helsinki Helsinki Finland; ^2^ Faculty of Medicine and Health Technology Tampere University Tampere Finland; ^3^ Indian Institute of Science Education and Research (IISER) Pune Pune Maharashtra India; ^4^ Fimlab Laboratories Tampere Finland

**Keywords:** biosensors, cell signaling, FLIM, fluorescent proteins, FRET, miRFP670nano3, mRuby2

## Abstract

Fluorescence resonance energy transfer (FRET) biosensors enable quantitative visualization of protein interactions and signaling dynamics in living cells. However, most fluorescent protein (FP) FRET pairs occupy overlapping spectral regions, limiting multiplexing and simultaneous imaging of multiple pathways. In particular, the limited availability of well‐characterized near‐infrared (NIR) acceptors has constrained robust dual‐FRET measurements within single cells. Here, we characterize a red‐to‐NIR FP FRET pair comprising mRuby2 and miRFP670nano3. Fluorescence lifetime imaging microscopy (FLIM) demonstrates efficient energy transfer with minimal spectral bleed‐through and compatibility with established CFP/YFP‐based reporters. In comparison with alternative dual‐FRET strategies, including dark acceptors and long Stokes shift FPs, this pair enables multiplexed imaging without extensive spectral unmixing or correction. Structural modeling suggests that favorable donor–acceptor geometry may contribute to its FRET efficiency. We demonstrate the utility of this red‐to‐NIR pair by simultaneous FLIM‐FRET imaging of Akt and S6K activity in single cells, providing parallel readouts of distinct branches of PI3K‐mTOR signaling. In three‐dimensional invasion assays, dual imaging of Src and ROCK reporters reveals spatially coordinated signaling dynamics not evident in single‐reporter measurements. Together, these results establish mRuby2/miRFP670nano3 as a practical addition to the FP‐FRET toolbox for multiplexed interrogation of signaling networks in living cells.

## Graphical abstract

Genetically encoded FRET biosensors reveal dynamic signaling in living cells, but overlapping spectra have limited the simultaneous monitoring of multiple pathways. We introduce a red‐to‐near infrared FRET pair, mRuby2/miRFP670nano3, compatible with existing reporters and enabling dual FLIM‐FRET without bleed‐through. This approach allows concurrent tracking of distinct kinase activities, uncovering dynamic behaviors such as front‐rear polarity cycling during cell invasion.

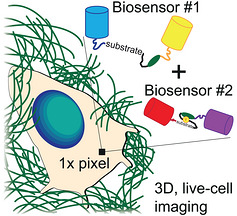

## 1 Introduction

Fluorescent protein (FP)‐based biosensors enable dynamic, non‐invasive imaging of molecular processes in living cells and tissues. Fluorescence resonance energy transfer (FRET) reporters provide a means to monitor protein‐protein interactions, conformational changes, and enzymatic activities in real time [1]. When combined with fluorescence lifetime imaging microscopy (FLIM), they support concentration‐independent measurements with high spatial and temporal resolution [1].

Most genetically encoded FRET reporters rely on FP pairs drawn from the multicolor GFP‐like protein superfamily [2, 3], composed of variants of cyan fluorescent protein (CFP) and yellow fluorescent protein (YFP), or green fluorescent protein (GFP) and one of a range of red fluorescent proteins (RFPs) [4, 5, 6, 7, 8]. Despite their widespread success, they are limited by spectral overlap, phototoxicity and crowding within the visible spectrum. These constraints are particularly restrictive for multiplexed or dual‐FRET imaging, where the limited spectral bandwidth of CFP/YFP and GFP/RFP variants hampers the simultaneous use of multiple reporters. While redshifted and near‐infrared (NIR) fluorescent proteins have expanded the available palette, red‐to‐NIR FRET combinations remain comparatively under‐characterized, and robust validation for general live‐cell FLIM applications is still limited [9, 10].

Recent advances in NIR FP engineering have begun to address these limitations. Notably, miRFP670nano3 [11, 12] (hereafter referred to as miRFP670) combines favorable photophysical properties with a compact size, making it an attractive candidate for use as a NIR FRET acceptor. NIR‐to‐NIR FRET pairs have been reported previously, including work employing the compact miRFP variants miRFP670nano [11, 13] and miRFP718nano [14], along with dual‐FRET multiplexed imaging with the larger bacterial photoreceptor BphP‐derived NIR FPs [9]. The availability of such NIR FP variants creates opportunities to develop and optimize FRET reporter pairs that expand the accessible spectral window and facilitate multiplexed imaging strategies. In this work, we characterize a red‐to‐NIR fluorescent protein FRET pair composed of mRuby2 and miRFP670 and evaluate its performance for live‐cell dual‐imaging applications beyond the spectral limits of conventional CFP/YFP‐ and GFP/RFP‐based reporters.

## 2 Results

### 2.1 mRuby2 is an Effective Donor for Red‐to‐NIR FRET to miRFP670

**FIGURE 1:**
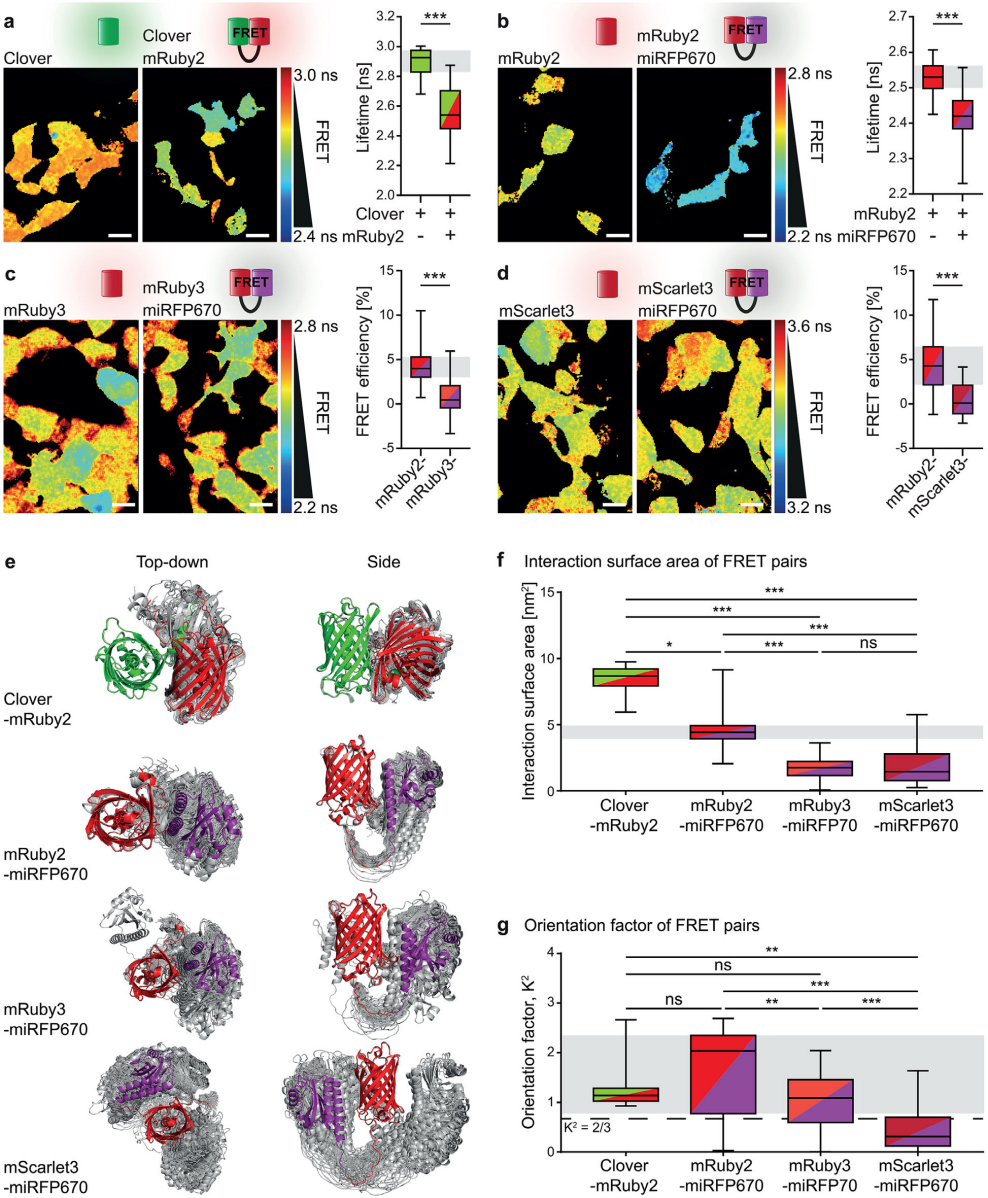
Red‐to‐NIR FRET can be achieved with mRuby2 paired with miRFP670. (a,b) Representative images (left) and fluorescence lifetimes (right) from HEK293FT cells transfected with (a, *n* = 3) Clover or Clover‐mRuby2, or (b, *n* = 5) mRuby2 or mRuby2‐miRFP670 (15‐25 cells/condition/replicate; *p*‐values from a Mann‐Whitney test; ^***^ *p* < 0.001; Scale bars, 20 µm). (c,d) Representative images (left) and FRET efficiencies (right) from HEK293FT cells transfected with mRuby2 (i.e. donor‐only control) or mRuby2‐miRFP670 compared to either (c) mRuby3 or mRuby3‐miRFP670, or (d) mScarlet3 or mScarlet3‐miRFP670 (*n* = 3 biological replicates, 20–27 cells/condition/replicate; *p*‐values from a Mann‐Whitney test; ^***^ *p* < 0.001; Scale bars, 20 µm). (e) AlphaFold3‐generated structures from top‐down and side view. All generated structures for the pairs are given in gray, with one representative structure colored. (f,g) Comparison of predicted (f) interaction surface area and (g) orientation factor for the given donor–acceptor pairs (100 predictions/pair; *p*‐values from a Kruskal‐Wallis test with a Dunn's correction for multiple comparisons; ^*^ *p* < 0.05, ^**^ *p* < 0.01, ^***^ *p* < 0.001; ns, not significant). Gray regions on boxplots indicate the interquartile range of the control condition. All boxplots include the median, interquartile range, and full data range (min‐to‐max whiskers).

To identify a suitable red‐to‐NIR FRET pair, the widely used fluorescent protein mRuby2 was first evaluated in a tandem construct with miRFP670. To confirm that mRuby2 was functional in our experimental system, we first verified its pairing with Clover, as previously described [4], observing a significant decrease in the fluorescence lifetime of Clover consistent with efficient FRET to mRuby2 (Figure 1A). We subsequently paired mRuby2 with miRFP670 and observed a significant decrease in fluorescence lifetime for mRuby2 (Figure 1B), confirming efficient red‐to‐NIR energy transfer. In contrast, performing the same experiment with RFPs developed more recently than mRuby2, namely mRuby3 and mScarlet3, revealed no detectable FRET when paired with miRFP670 (Figure 1C, mRuby3; Figure 1D, mScarlet3).

To further investigate this discrepancy, we assessed the relative fluorescence intensity of mRuby2, mRuby3, and mScarlet3 expressed alone or tethered to miRFP670, and observed no loss of fluorescence that could account for the absence of FRET with mRuby3 or mScarlet3 as donors, arguing against protein unfolding or destabilization due to the closer proximity of miRFP670 (Figure S1A,B). Moreover, co‐expression of miRFP670 with mRuby2 did not alter the fluorescence lifetime of mRuby2 (Figure S1C), confirming that mRuby2 and miRFP670 form an effective FRET pair and that the observed lifetime changes are not due to direct perturbation of mRuby2 fluorescence by bleed‐through from miRFP670. Additionally, sequential analysis of mRuby2 fluorescence lifetimes across four consecutive 150‐frame acquisition segments revealed no significant variation over time (Figure S1D), demonstrating that previously reporter photochromic dark‐state transitions [15, 16] do not measurably affect donor lifetime under our experimental conditions. Furthermore, when we performed in silico assessment of the interaction of mRuby2, mRuby3 and mScarlet3 with miRFP670, we observed mRuby2 to interact with miRFP670 with a larger contact area (4.5 ± 1.2 nm^2^ vs. 1.8 ± 0.8 and 2.0 ± 1.5, respectively) with a more defined binding pose as compared to other FRET pairs (Figure 1E,F). This is consistent with the observed increase in FRET resultant from modification of the dimerization interface between ECFP/EYFP to ECFP/YPet [4, 5, 17], and between Clover/mCherry and Clover/mRuby2 [4, 18], further supported by our observation of strong complexation between those improved FRET pairs (Figure S1E,F). We also observed a higher predicted orientation factor distribution for mRuby2 compared to mRuby3 and mScarlet3 (1.7 ± 0.8 vs. 1.0 ± 0.5 and 0.4 ± 0.4, respectively) (Figure 1G), which together with a larger interaction surface area suggests that the mRuby2/miRFP670 pair could have a greater tendency to form donor–acceptor interactions that guide them into a more favorable orientation for higher FRET. Overall, in silico modeling of the pairs suggests that the observed increase in FRET between mRuby2 and miRFP670 might arise from an enhanced interaction interface and more favorable chromophore orientation upon association. We acknowledge that the flexible (GGGGS)_3_ linker used in these constructs permits a wide range of donor–acceptor orientations in principle, and that FRET does not require stable protein‐protein interaction or direct contact. However, if preferential surface interactions cause the donor and acceptor to adopt a favored relative orientation, the chromophore dipoles will not sample all possible angles equally, and the measured FRET efficiency will reflect this preferred geometry rather than the random tumbling assumed by the standard κ^2^ = 2/3 approximation [19, 20].

We further note that the effective local concentration of tethered donor and acceptor domains is intrinsically high regardless of linker length: under a random coil model, a 50‐residue flexible linker corresponds to an estimated effective concentration of ∼13 mm (see section 4, Methods), far exceeding what is achievable with separate proteins at any practical bulk concentration, rendering transient contacts kinetically accessible even in the absence of a strong binding interface.

This interpretation is consistent with deliberate engineering of interaction interfaces in other FRET pairs to have better contacts [21, 22]. Importantly, the proposed interaction is expected to be weak and transient rather than constitutive, which is consistent with the observation that weakly dimerizing FP variants yield the greatest biosensor dynamic range, while strongly dimerizing variants collapse the FRET response by locking the construct in a high‐FRET ground state [23]. These structural features are diminished in mRuby3 and not predicted in mScarlet3, consistent with their lack of detectable FRET. We note that AlphaFold models represent predicted low‐energy docking poses rather than a full sampling of the conformational ensemble accessible to flexibly tethered FP pairs in a cellular environment [24]. These models are therefore presented as a structural foundation for the observed differences between FP pairs rather than a quantitative description of the FRET geometry in cellulo.

### 2.2 Comparison to Previous Dual FLIM‐FRET Approaches Highlights the Clear Advantages of mRuby2/miRFP670

**FIGURE 2:**
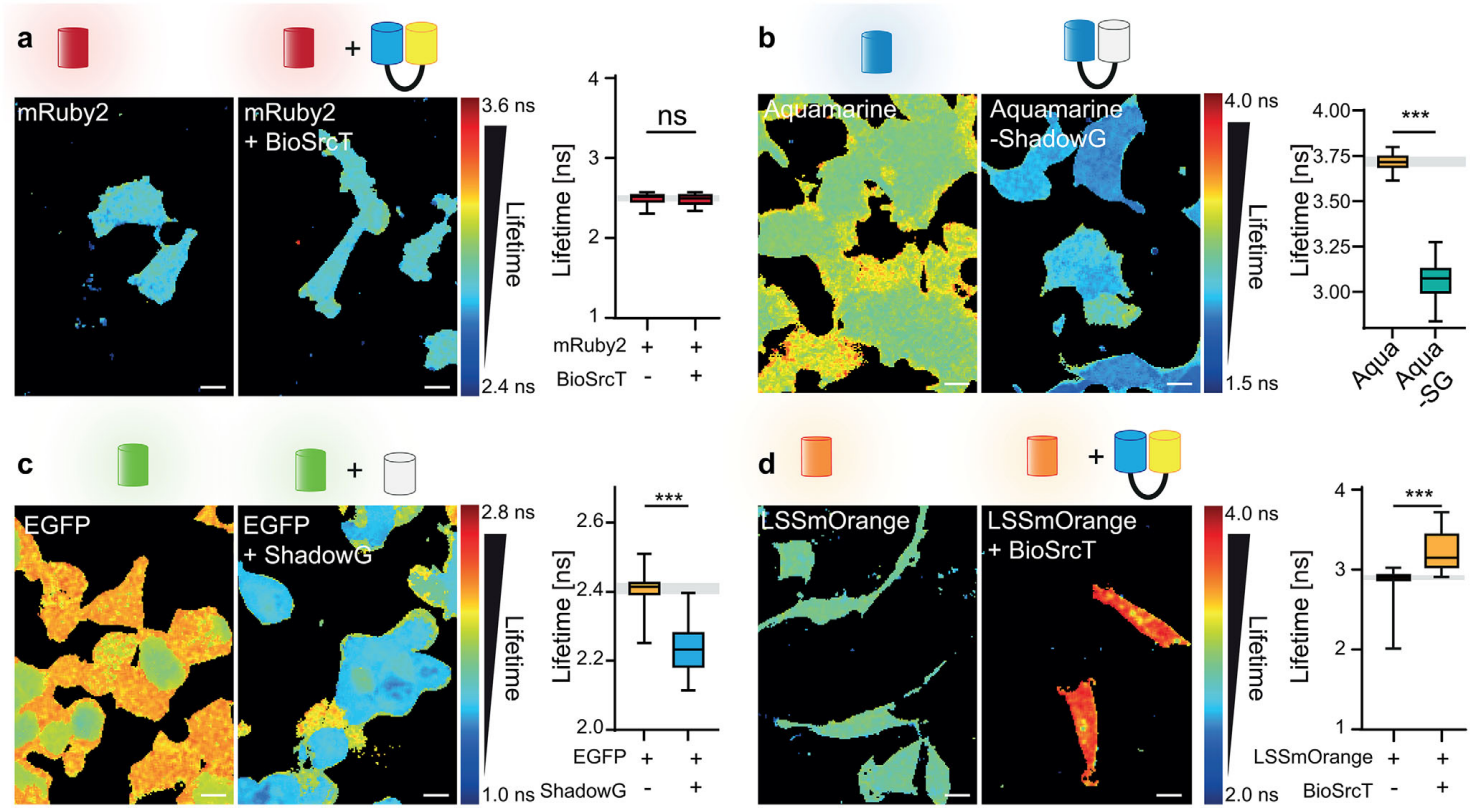
Assessment of spectral bleed‐through for dual FLIM‐FRET applications. (a) Representative images (left) and fluorescence lifetimes (right) from HEK293FT cells transfected with mRuby2 or both mRuby2 and BioSrcT (*n* = 3, 16–23 cells/condition/replicate; *p*‐values from a Mann‐Whitney test; ns, not significant; Scale bars, 20 µm). (b) Representative images (left) and fluorescence lifetimes (right) from HEK293FT cells transfected with either Aquamarine (Aqua, i.e., donor‐only control) or Aquamarine‐ShadowG (Aqua‐SG, *n* = 3 biological replicates, 20–31 cells/condition/replicate; p‐values from a Student's t‐test with Welch's correction; ^***^ *p* < 0.001; Scale bars, 20 µm). (c) Representative images (left) and fluorescence lifetimes (right) from HEK293FT cells transfected with EGFP or both EGFP and ShadowG (14‐20 cells/condition/replicate; *p*‐values from a Mann‐Whitney test; ^***^ *p* < 0.001; Scale bars, 20 µm). (d) Representative images (left) and fluorescence lifetimes (right) from HEK293FT cells transfected with LSSmOrange or both LSSmOrange and BioSrcT (*n* = 3, 21–25 cells/condition/replicate; *p*‐values from a Mann‐Whitney test; ^***^ *p* < 0.001; Scale bars, 20 µm). Gray regions on boxplots indicate the interquartile range of the control condition. All boxplots include the median, interquartile range, and full data range (min‐to‐max whiskers).

To assess whether this pair could support simultaneous imaging of two FRET reporters in the same cell, mRuby2 was transfected into cells expressing the BioSrcT FRET reporter, which utilizes the mTurquoise2/YPet FRET pair. Under these conditions, we observed no significant difference in either the fluorescence lifetime (Figure 2A) or channel intensity (Figure S2A) of mRuby2 in cells expressing BioSrcT compared with controls, supporting the compatibility of the mRuby2/miRFP670 FRET pair with reporters based on CFP/YFP variants.

Previous approaches to enable the co‐expression of two FRET reporters have addressed spectral bleed‐through using dark acceptors, in which a CFP variant undergoes efficient FRET to a non‐fluorescent acceptor without restricting the use of GFP/RFP‐based reporters [25, 26]. Consistent with these studies, we detected a reduced fluorescence lifetime when Aquamarine was tethered to ShadowG (Figure 2B), supporting the suitability of this pair for lifetime‐based FRET measurements. However, when EGFP was co‐expressed with ShadowG, we observed a significant reduction in the measured lifetime (Figure 2C), attributable to spectral bleed‐through of the short‐lifetime ShadowG emission into the donor detection channel. It is thus difficult to ascertain whether this decrease is arising from true energy transfer or instead reflects contamination of the donor channel with acceptor bleed‐through. These observations suggest that, while dark acceptors provide an elegant solution to spectral overlap, their use in dual‐FLIM experiments may require additional controls or fitting strategies to accurately separate the contributions of different FRET pairs and reliably extract the donor‐specific lifetime.

An alternative strategy for dual‐FRET imaging has employed long Stokes shift orange fluorescent proteins, such as LSSmOrange, which functions as an effective donor for mKate2 (Figure S2B) [27]. However, when LSSmOrange was co‐expressed with the BioSrcT reporter, we observed substantial bleed‐through arising from the CFP/YFP variants into the LSSmOrange detection channel, evidenced by both increased channel intensity (Figure S2C) and a confounding shift in fluorescence lifetime (Figure 2D), with phasor analysis further confirming that BioSrcT signal directly distorts LSSmOrange lifetime interpretation (Figure S2D). This is consistent with previous reports in which this limitation was addressed through pairing LSSmOrange with a dark acceptor, such as ShadowG paired with mTFP1 [28]. In that configuration, LSSmOrange fluorescence lifetimes remained stable across a range of expression conditions when ShadowG was used as the acceptor, indicating effective suppression of donor bleed‐through into the orange channel, whereas low‐level lifetime perturbations were detected with the less dark acceptor sREACh under similar conditions [28]. Ongoing development of improved dark acceptor variants, including ShadowY [29], may further mitigate such residual lifetime effects and bleed‐through‐related artefacts; nevertheless, careful validation remains necessary to ensure that observed lifetime changes reflect genuine donor modulation rather than bleed‐through.

Importantly, these constraints are effectively avoided with the use of the mRuby2/miRFP670 FRET pair, which enables dual FLIM‐FRET imaging without detectable bleed‐through between channels under our imaging conditions, as supported by both intensity‐based and lifetime‐based measurements and is compatible with a wide range of existing CFP/YFP‐based reporters.

### 2.3 Co‐Expression of FRET Reporters for Both S6K and Akt Allows Tracking of Distinct Signaling Components of the PI3K Pathway

**FIGURE 3:**
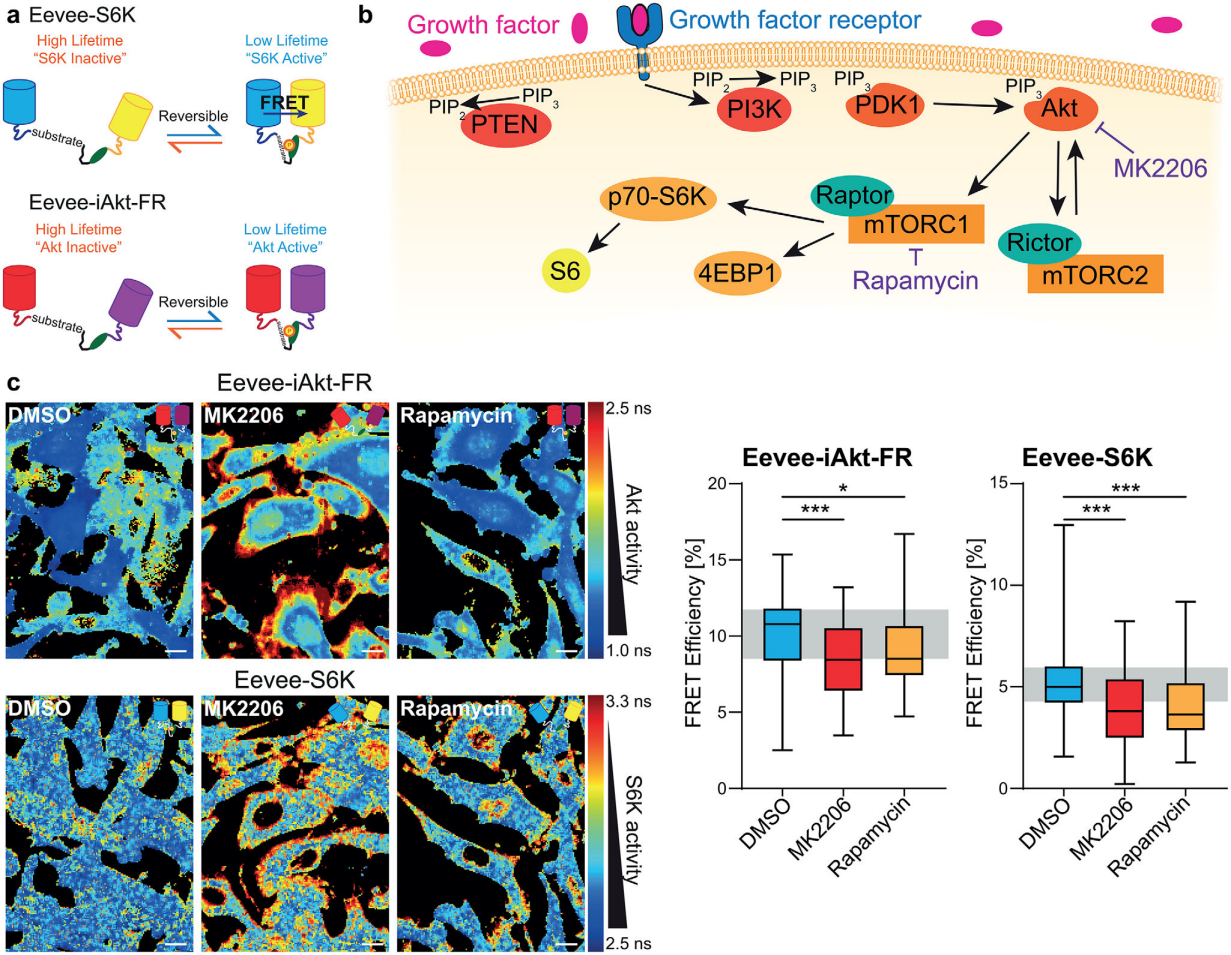
Dual FLIM‐FRET demonstrates PI3K pathway‐specific modulation of activation states. (a) Schematic of the Eevee‐S6K and Eevee‐iAkt‐FR FRET reporters, where the FHA1 phosphorylation binding domain is given in green. (b) PI3K pathway scheme highlighting the distinct targets of rapamycin and MK2206. (c) Representative images (left) and FRET efficiencies (right) from MDA‐MB‐231 (MM231) cells stably expressing both Eevee‐iAkt‐FR and Eevee‐S6K and treated with either rapamycin (100 nm) for 2 h, or MK2206 (500 nm) or DMSO control for 16 h (*n* = 3, 20–68 cells/condition/replicate; *p*‐values from a Kruskal–Wallis test with a Dunn's correction for multiple comparisons; ^*^ *p* < 0.05, ^***^ *p* < 0.001; FRET efficiency calculated using “donor‐only” MM231s transfected with mTurquoiseGL or mRuby2; Scale bars, 20 µm). Gray regions on boxplots indicate the interquartile range of the control condition. All boxplots include the median, interquartile range, and full data range (min‐to‐max whiskers).

To test the feasibility of dual FLIM‐based imaging of two FRET reporters within the same cells, we co‐expressed Eevee‐S6K (mTurquoiseGL/YPet FRET pair) together with a NIR‐shifted Eevee‐iAkt (mRuby2/miRFP670 FRET pair), hereafter referred to as Eevee‐iAkt‐FR (Figure 3A). To minimize recombination between the highly similar mTurquoiseGL and YPet sequences (similarity >95%), Eevee‐S6K was first introduced using the PiggyBac transposon system [30, 31], followed by lentiviral transduction of the NIR reporter, which employs a mRuby2/miRFP670 FRET pair with low sequence homology (similarity < 45%).

The S6K reporter contains a substrate sequence derived from Rictor, a core component of mTOR complex 2 (mTORC2; Figure 3B) that is directly phosphorylated by S6K1 downstream of mTORC1. This design allows the reporter to detect mTORC1‐dependent S6K1 activity, including signaling events that are sensitive to rapamycin and occur independently of Akt phosphorylation at Ser473 [32, 33]. In contrast, FRET reporters for Akt activity have undergone multiple iterations to improve their dynamic range, including the introduction of an extended linker (i.e., Eevee linker) to stabilize the off‐state, as well as incorporation of the Akt PH domain to enhance subcellular localization, together resulting in improved reporter performance [34]. Subsequent optimization introduced a substrate derived from the Akt target GSK3β [35]; however, we observe only a modest increase in reporter sensitivity with this sequence (Figure S3A; compare the mean rank difference of Eevee‐Akt‐mT2, 64.2, with 78.9 for Eevee‐iAkt‐mT2).

Importantly, replacement of the original FRET pair with mRuby2/miRFP670 preserves sensitivity to Akt inhibition downstream of mTORC2, while enabling concurrent tracking of S6K activity downstream of mTORC1 within the same cells (Figure 3C). Indeed, the treatment‐induced change in FRET efficiency following MK2206 treatment was similar between reporters; 1.14% ± 0.29% for Eevee‐iAkt‐mT2 (Figure S3A; mean ± SD) and 1.29% ± 1.87% for Eevee‐iAkt‐FR (Figure 3C; mean ± SD). This was associated with similarly significant changes in the population‐level phosphorylation state of Akt(Ser473) and S6(Ser235/Ser236) (Figure S3B). Previous studies using the Akt PH domain in reporters based on EGFP variants reported poor plasma membrane localization [34, 36], an effect attributed to the strong negative electrostatic charge of EGFP‐derived FPs [37]. However, substitution with the weakly charged mRuby2/miRFP670 FRET pair did not resolve this localization defect (Figure S3C,D), suggesting that further optimization of electrostatic charge in next‐generation red‐to‐NIR FRET pairs may provide additional benefits beyond improved FRET efficiency.

### 2.4 Dual FLIM‐FRET of ROCK and Src Kinase in Invading Breast Cancer Cells Highlights Spatiotemporal Dynamics

**FIGURE 4:**
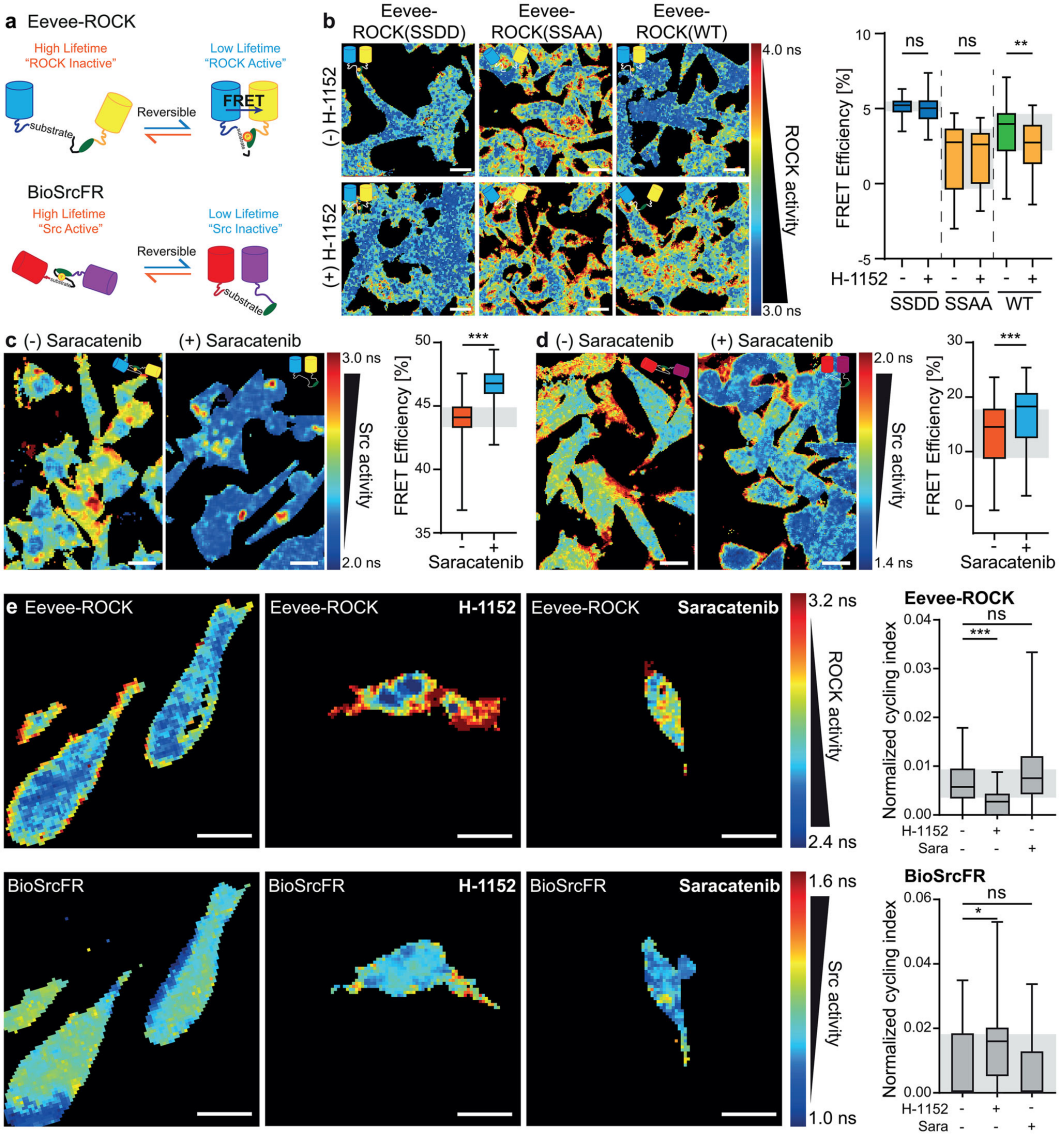
Live tracking of ROCK and Src kinase activity during cancer invasion. (a) Schematic of the Eevee‐ROCK and BioSrcFR FRET reporters, where the phosphorylation‐binding domains are given in green (FHA1 for Eevee‐ROCK and an SH2 domain for BioSrcFR). (b) Representative images (left) and FRET efficiencies (right) from MM231 cells stably expressing either Eevee‐ROCK(WT/SSDD/SSAA) and treated with H‐1152 (5 µm) or DMSO for 2 h (*n* = 3 biological replicates, 23–26 cells/condition/replicate; *p*‐values from a Kruskal–Wallis test with a Dunn's correction for multiple comparisons; ns, not significant; ^**^ *p*<0.01; FRET efficiency calculated using “donor‐only” MM231 cells transfected with mTurquoiseGL; scale bars, 20 µm). (c,d) Representative images (left) and FRET efficiencies (right) from MM231 cells stably expressing either BioSrcT (c; *n* = 3 biological replicates, 61–71 cells/condition/replicate), or BioSrcFR (d; *n* = 4 biological replicates, 26–38 cells/condition/replicate) and treated with Saracatenib (1 µm; Sara) or DMSO for 24 h (*p*‐values from Mann–Whitney tests; ^***^ *p*<0.001; FRET efficiency calculated using “donor‐only” MM231 cells transfected with mTurquoise2 or mRuby2; scale bars, 20 µm). (e) Representative images (left) and normalized cycling indices (right) from MM231 cells stably expressing both Eevee‐ROCK and BioSrcFR and treated with H‐1152 (5 µm) for 2 h, or Saracatenib (1 µm; Sara) or DMSO for 24 h (*n* = 4 biological replicates, 2–26 cells/condition/replicate; *p*‐values from a Kruskal–Wallis test with a Dunn's correction for multiple comparisons; ns, not significant; ^**^ *p*<0.01, ^*^ *p*<0.05; scale bars, 20 µm). Gray regions on boxplots indicate the interquartile range of the control condition. All boxplots include the median, interquartile range, and full data range (min‐to‐max whiskers).

Having validated the effectiveness of the dual FLIM‐FRET approach, we next assessed live spatiotemporal signaling dynamics during cancer cell invasion in 3D. We first developed a FRET reporter for ROCK based on the Eevee backbone [34], with the substrate sequence modified to that of MLC2/*MYL2* (Figure 4A). Expression of this reporter in HEK293FT (Figure S4) or MDA‐MB‐231 (MM231) breast cancer cells (Figure 4B) revealed a reduction in reporter phosphorylation upon ROCK inhibition, which was not observed with phosphorylation‐defective (SSAA) or phosphorylation‐mimetic (SSDD) mutants.

We next generated a red‐shifted Src activity reporter based on previously described constructs [38, 39, 40], which exhibited the expected decrease in Src activity following inhibition (Figure 4C,D; treatment‐induced change of 2.60% ± 1.29% for BioSrcT or 3.85% ± 0.98% for BioSrcFR (mean ± SD)). Cells co‐expressing the ROCK and Src FRET reporters were then embedded in a 3D extracellular matrix contracted by unlabeled fibroblasts to generate an in vivo‐like environment for monitoring invasion [41].

Strikingly, ROCK activity exhibited robust front‐rear cycling, whereas Src activity showed little evidence of cycling under control conditions (Figure 4E; Movies S1–S6). Cycling was quantified using a normalized cycling index, defined as the fraction of time intervals in which front‐rear polarity reversed sign, scaled by the variability of the polarity signal to account for amplitude differences and suppress noise‐driven fluctuations. With this approach, we observed that inhibition of ROCK abolished ROCK cycling and led to increased Src cycling, whereas Src inhibition had no detectable effect on the dynamics of either pathway. Importantly, validation in 2D demonstrated that this occurred without dissociation of either reporter by proteolytic cleavage or degradation (Figure S4B) and arose with similarly significant changes in the population‐level phosphorylation state of MLC2(Ser19) (Figure S4C). Together, these results reveal a hierarchical organization of invasive signaling dynamics in which ROCK‐dependent contractility governs front‐rear polarity cycling, while Src activity is dynamically constrained and becomes labile only upon disruption of ROCK signaling. The work also demonstrates the power of dual FLIM‐FRET imaging for the dissection of signaling hierarchies that are obscured in single‐reporter or population‐averaged analyses.

## 3 Conclusion

In this study, we identify and validate a red‐to‐NIR FRET pair composed of mRuby2 and miRFP670, and demonstrate its utility for live dual FLIM‐FRET in 2D and 3D systems. We show that mRuby2 undergoes efficient FRET to miRFP670, while closely related RFPs fail to support red‐to‐NIR FRET. Based on computationally predicted protein‐protein complexes, we propose that this reflects the capacity of mRuby2 to interact better with miRFP670, supported by the observation that several previously described FRET pairs appear to form more extensive donor–acceptor interactions compared to reportedly poor FRET pairs (Figure S1E,F). Previous studies have already highlighted the utility of deliberately optimizing the interaction interface between a FP pair to increase the FRET efficiency [20, 21, 22, 23, 42]. Importantly, this FRET pair is compatible with existing CFP/YFP‐based reporters, enabling dual FLIM‐FRET measurements without detectable spectral bleed‐through. Using this approach, we simultaneously monitored Akt and S6K activities within single cells, allowing parallel interrogation of distinct branches of the PI3K‐mTOR signaling network. While S6K activity is classically associated with mTORC1, it is also influenced by mTORC2‐dependent inputs. Thus, the combined readout of Akt and S6K enables discrimination between mTORC1‐ and mTORC2‐associated signaling, including rapamycin‐sensitive and ‐insensitive mTORC2 outputs, a level of pathway resolution that cannot be achieved using a single biosensor alone.

Genetically encoded FRET reporters are widely used to probe dynamic signaling processes in live cells, providing high temporal resolution and quantitative readouts of molecular activity. When combined with FLIM, these reporters offer concentration‐independent measurements that are particularly valuable in heterogeneous or dynamic environments [40]. However, the reliance on CFP/YFP or GFP/RFP variants has constrained multiplexing and limited their applicability for intravital or deep‐tissue imaging, where autofluorescence, scattering, and spectral crowding compromise signal fidelity, which is circumvented when applying NIR FPs [12]. Alternative approaches such as hyperspectral FRET imaging can partially alleviate spectral overlap, but require specialized instrumentation and computational unmixing [43]. These limitations can be mitigated by the use of NIR fluorescent proteins.

Expanding on the applications of these NIR FPs, we demonstrate their use in dissecting spatiotemporal signaling dynamics during cancer cell invasion in a 3D extracellular matrix. By combining newly developed FRET reporters for ROCK and Src activity, we reveal distinct and dynamic behaviors that are not apparent from single‐reporter measurements. ROCK activity exhibited pronounced front‐rear cycling during invasion, whereas Src activity remained comparatively stable under basal conditions. Perturbation experiments uncovered a hierarchical organization in which ROCK‐dependent contractility governs polarity cycling and constrains Src dynamics, with Src becoming labile only upon disruption of ROCK signaling. Together, these findings highlight how simultaneous pathway monitoring using multiplexed FLIM‐FRET enables resolution of signaling hierarchies and functional architectures that are otherwise obscured in population‐averaged or single‐reporter analyses.

A practical consideration for users is the difference in chromophore maturation between mRuby2 and miRFP670. GFP‐like RFPs such as mRuby2, derived from eqFP611, undergo autocatalytic chromophore formation requiring protein folding, backbone cyclization, dehydration and oxidation, with a reported half‐time of ∼2.8 h at 37°C in vitro for the parental mRuby [44], and ∼150 min for mRuby2 [4]. In contrast, miRFP670 and related cyanobacteriochrome‐derived NIR FPs mature by covalent incorporation of biliverdin IXα, a heme catabolite constitutively available in mammalian cells [13]. This cofactor‐dependent maturation mechanism, with a half‐time of ∼100 min reported for miRFP670nano [13], proceeds independently of molecular oxygen and is therefore less sensitive to hypoxic conditions than GFP‐like FPs. In practical terms, both proteins are expected to be substantially mature within a few hours of expression under standard cell culture conditions, and the maturation difference is unlikely to pose a significant limitation for steady‐state imaging applications. However, for experiments involving inducible or pulse‐chase expression, such as tracking of newly synthesized protein pools or monitoring of rapid signaling events, the kinetic offset between mRuby2 and miRFP670 maturation should be taken into account when interpreting FRET signals, as a transient population of immature donor molecules may confound measurements.

Despite these advances, further optimization of red‐to‐NIR FRET reporters remains an important goal. The dynamic range of FRET sensors is ultimately limited by donor and acceptor brightness, orientation, interaction, and photostability. Continued engineering of NIR fluorescent proteins with increased quantum yield, reduced charge‐related localization artifacts and improved photophysics are likely to yield further improvements in FRET efficiency and biosensor performance. Our findings pinpoint donor–acceptor interactions as an important target for further optimization. In this way, compact NIR FP variants derived from the same miRFPnano family, such as miRFP704nano [14] and miRFP718nano [45], as well as other alternative NIR acceptors [12], represent promising candidates for evaluation in this context. Such developments may enable next‐generation red‐to‐NIR FRET pairs with enhanced dynamic range and even broader applicability for in vitro and intravital imaging.

## 4 Methods

### 4.1 Cell Lines

MM231 (HTB‐26, ATCC) and HEK293FT (R70007, ThermoFisher) cells, along with Human dermal fibroblasts, TIFs (a kind gift from J.C. Norman (Beatson Institute, Glasgow, Scotland, UK)), were maintained in Dulbecco's modified Eagle's medium (DMEM; Sigma, D5796) supplemented with 10% serum (FBS; Biowest, S181T), L‐glutamine (100 mm, Sigma, G7513), HEPES (10 mm, Sigma, H0887) and sodium pyruvate (1 mm, Sigma, S8636).

### 4.2 Computational Analysis and Modelling of FRET Pairs

FRET pairs were generated using AlphaFold3 server [24], with a GGGGSx3 linker included between the donor and acceptor sequences. For FRET pairs consisting of miRFP670 together with various RFPs, we generated 100 (20 × 5) structural models, consisting of 20 inputs that result in 5 different predictions each. This was done with template usage disabled to reduce the biasing of the system with existing models. For reference pairs shown in the Supporting Information, we generated 25 structures (5 × 5).

Interaction surface area was calculated from solvent accessible surface area (SASA) using GROMACS 2025 [46, 47] with the gmx sasa command and Equation (1):

(1) $$\begin{array}{c} Interaction\;sur\;f\;ace\;area=\\ \frac{SASA\left(donor\right)+SASA\left(acceptor\right)-SASA\left(complex\right)}{2} \end{array}$$

To evaluate the FRET‐capability of the pairs based on their orientation in the docked configuration, transition dipole moment (TDM) and center of mass (CoM) for donor and acceptor chromophores were determined as presented in Teijeiro‐Gonzalez, Y. et al. [48], selecting the same atomic positions for all chromophores, except for miRFP670's chromophore (RCSB PDB: JRA). For JRA, TDM was calculated using ORCA [49, 50] time‐dependent density functional theory (TDDFT) calculation with B3LYP, def2‐SVP, and TightSCF parameters. TDM orientation was confirmed by comparing to a highly similar structure with known TDM [51]. Selections for TDM vector and CoM were done similarly than for other studied chromophores to best approximate the orientation of the TDM and CoM of the molecule [48], and are presented in Figure S5. Orientation factor was calculated using Equation (2):

(2) $$\kappa^{2}={\left(\cos\alpha_{DA}-3\cos\alpha_{D}\cos\alpha_{A}\right)}^{2}$$

Values for α_*DA*_, α_*D*_ and α_*A*_ were obtained with Visual Molecular Dynamics (VMD; version 1.9.3) [52]. PyMOL (Version 3.0, Schrödinger) was used to visualize structures.

### 4.3 Confocal Imaging

MM231 cells were transfected with the indicated plasmids using Lipofectamine3000 (ThermoFisher, L3000), as per the manufacturer's protocol. Imaging was performed using a Leica Stellaris DMi8 inverted microscope with an HC PL APO CS2 63X/1.2 water immersion objective and a pinhole set to 1 Airy unit. For mRuby2, mRuby3 and Scarlet3, the white light laser was tuned to 561 nm for excitation, and emission was detected from 570–610 nm on a HyD S detector. For miRFP670, the white light laser was tuned to 633 nm for excitation, and emission was detected from 644–751 nm on a HyD S detector. For LSSmOrange, the white light laser was tuned to 448 nm for excitation, and emission was detected from 560–580 nm. For the bleed‐through controls with BioSrcT, excitation by the white light laser was at either 448 nm (for LSSmOrange co‐transfection) or 488 nm (for mRuby2 co‐transfection), with emission detected at 461–500 or 500–540 nm respectively. Frame averaging was performed from a total of 8 frames. The fluorescence ratio was calculated from the fluorescence intensity per cell extracted from Fiji (NIH) and plotted as a ratio between the mRuby2, mRuby3, or mScarlet3 and the co‐transfected miRFP670.

### 4.4 Electrostatic Potential Calculations

Protein structures for miRFP670 (PDB 7LSC), mTurquoise2 (PDB 6YLN), and the PH domain of Akt2 (PDB 1P6S) were prepared and pKa values calculated using the PDB2PQR webserver with default parameters [53]. Electrostatic potentials were subsequently determined by solving the Poisson‐Boltzmann equation via the APBS webserver [53], as achieved previously [37]. For mRuby2 and YPet, predicted structural models were first generated using AlphaFold3 [24] before applying the same PDB2PQR/APBS workflow. The resulting structures and electrostatic maps were visualized in ChimeraX, version 1.10.1 (2025‐07‐24) [54].

### 4.5 Estimation of Effective Local Concentration for a Flexible Peptide Linker Using a Random Coil Model

Flexible peptide linkers in solution typically behave as random coils, whose average dimensions follow polymer scaling laws (*R* ∼ *N* ^1/2^) characteristic of disordered polypeptides [55, 56, 57]. In such a case, the end‐to‐end distance (R) can be approximated using Equation (3), where R = end‐to‐end distance, a = Kuhn length (effective segment length), and *N* = number of residues:

(3) $$R∼a\cdot \sqrt{N}$$

Therefore, a 50‐residue flexible linker is expected to span ∼3–5 nm depending on the assumed Kuhn length (0.7–1.0 nm), consistent with experimental measurements of disordered polypeptides. Using a typical statistical segment length of 0.7 nm we thus obtain an average end‐to‐end distance for a 50‐residue flexible linker with Equation (4):

(4) $$R\approx 0.7\times \sqrt{50}\approx 0.7\times 7.1\approx 5\;\mathrm{nm}$$

To estimate the effective concentration of solution where the average distance between molecules is 5 nm, we can then use Equation (5):

(5) $$\begin{array}{ccc} n∼d^{-3}={\left(5\;\times \;10^{-9}m\right)}^{-3} & & =\frac{1}{125\;\times \;10^{-27}}molecules/m^{3}=\\ & & 8\;\times \;10^{24}molecules/m^{3} \end{array}$$

This divided by Avogadro's number 6.022 × 10^23^ mol^−1^ provides an effective concentration of 13.3 mm.

### 4.6 Time‐Domain FLIM‐FRET

All FLIM experiments were performed on a Leica Stellaris DMi8 inverted microscope with a FALCON FLIM module. A HC PL APO CS2 motCORR 63X/1.2 water was used for all experiments except for those with BioSrcT, which used a HC PL APO CS2 20X/0.75 dry objective. H‐1152 (HY‐15720A), rapamycin (HY‐10219), MK‐2206 (HY‐10358) and Saracatenib (HY‐10234) were all ordered from MedChemExpress as 1 mm stocks in DMSO. Fixation was performed in 4% paraformaldehyde for 10 min at 37°C, followed by quenching in glycine (1 m, Sigma–Aldrich, G7126) and storage in PBS to avoid disruption of lifetime measurements [58]. For experiments with HEK293FT cells, the plasmids were transiently transfected, while experiments conducted with MM231 cells were performed with stably expressing cell lines. For the mRuby2, mRuby3 and Scarlet3 FRET controls and constructs, the white light laser was tuned to 561 nm for excitation, and emission was detected from 580–610 nm on a HyD X detector. No detectable fluorescence from miRFP670 was observed in the red detection channel (561 nm excitation) used for RFP lifetime measurements (Figure S1B), confirming the absence of acceptor bleed‐through into the donor channel. For the LSSmOrange FRET controls and constructs, the white light laser was tuned to 448 nm for excitation, and emission was detected from 560–580 nm on a HyD X detector. For the EGFP bleed‐through experiment with ShadowG, the white light laser was tuned to 488 nm for excitation, and emission was detected from 500–530 nm on a HyD X detector. For the Aquamarine, mTurquoise2 and mTurquoiseGL FRET controls and constructs, the white light laser was tuned to 448 nm for excitation, and emission was detected from 455–480 nm on a HyD X detector.

Lifetimes were calculated in the LAS X software (4.9.0.30221). While purified mRuby has been reported to exhibit a near‐monoexponential decay [44], multi‐exponential fluorescence decays are commonly observed for fluorescent proteins in cellular environments, arising from chromophore conformational heterogeneity and interactions with the cellular milieu [59, 60]. Consistent with this, a three‐exponential model provided a significantly better fit for the in cellulo FLIM data than one‐ or two‐exponential models, as assessed by χ^2^ and the structure of the residuals for the examples given of mRuby2 (Figure S6) and Clover (Figure S7). The individual lifetime components were not assigned to specific molecular species but served as empirical fitting parameters for the intensity‐weighted mean lifetime, which is robust to the precise number of components and provided the most stable and reproducible readout for all subsequent FRET efficiency calculations [61]. FLIM acquisitions were performed at 400 Hz (Pixel Dwell Time 2.825 µs) for 150–200 frames for fixed imaging regions of interest and 600 frames for assessment of photochromic dark‐state behavior in mRuby2, where the latter were subsequently divided into four sequential 150‐frame segments for longitudinal lifetime assessment. Phasor plots were generated using the LAS X software (4.9.0.30221) using the first Fourier harmonic of the fluorescence decay, a Wavelet filter for noise reduction, and a minimum photon count threshold of 5 per pixel. Representative lifetime maps were generated with FLIMfit (v5.1.1) [62], using gold nanoparticles (10 nm diameter, OD 1, suspension in 0.1 mM PBS, Sigma–Aldrich, 752584) to obtain the instrument response functions. The apparent FRET efficiency (*E_app_*) was calculated using the measured lifetimes of each donor–acceptor pair (τ_*DA*_) and the average lifetime of the donor‐only (τ_*D*_) samples, as in Equation (6):

(6) $$E_{app}=\left(1-\tau_{DA}/\tau_{D}\right)\times 100$$

### 4.7 Lentiviral Transduction

To generate MM231s with two FRET reporters, lentivirus was produced in HEK293FT cells by co‐transfecting with a third‐generation lentiviral packaging system composed of pMDLg/pRRE, pRSV‐Rev, pMD2.G, along with the pLenti6.3‐BioSrcFR or ‐Eevee‐iAkt‐FR transfer plasmids, using Lipofectamine3000 in OptiMEM (Gibco, 21985070), as per the manufacturer's protocol. The media was changed for complete growth medium after 24 h, further incubating for 24 h before collection and filtering through a 0.45 µm syringe filter. Cells were then transduced with lentivirus for 48 h in the presence of polybrene (8 µg/mL; Sigma, TR‐1003‐G), before washing and selecting stable positive cells using blasticidin (10 µg/mL; Merck, SBR00022).

### 4.8 Live Imaging of Invading Cancer Cells

Fibroblast‐contracted 3D collagen I matrices were generated as described previously [41, 63]. While TIFs were used to contract the matrices, the addition of MM231s stably expressing BioSrcFR and Eevee‐ROCK(WT) at the embedding stage meant that after 14 days of contraction, the matrices could be removed from the 6‐well plates and cut to fit into 8‐well Ibidi dishes for live imaging. Ascorbic acid (1 mm) was added to the media to reduce the effects of phototoxicity during imaging [64]. Images were acquired on a Leica Stellaris DMi8 inverted microscope with a FALCON FLIM module and a HC PL APO CS2 40x/1.25 glycerol‐immersion objective, tuning the white light laser to 448 nm for excitation of Eevee‐ROCK and 561 nm for BioSrcFR, emission was detected from 455–480 nm (Eevee‐ROCK) or 570–610 nm (BioSrcFR) on a HyD X detector for 8 frames/timepoint acquired at 400 Hz (Pixel Dwell Time 2.8 µs). Live imaging was performed at 37°C and 5% CO_2_ with image channels captured sequentially. The normalized cycling index was calculated for each cell using consecutive time points, where a polarity switch was scored when the front‐rear polarity values changed sign. The index was defined as the fraction of time intervals exhibiting a polarity switch, normalized by the median absolute deviation (MAD) of the polarity signal to account for differences in signal amplitude; values within a dead‐zone threshold (|polarity| < 5) were set to zero to reduce the effect of noise‐driven switches.

### 4.9 Molecular cloning

Controls for the assessment of mRuby2 and miRFP670 FRET efficiency were engineered by first restriction digestion of pcDNA3.1‐Clover‐mRuby2 with BamHI/KpnI and a gene block containing miRFP670nano3, where ligation yielded pcDNA3.1‐miRFP670nano3‐mRuby2. For the mScarlet3 variant, PCR was performed using pmScarlet3‐C1, followed by digestion with BamHI/EcoRI and ligation into the similarly digested pcDNA3.1‐miRFP670nano3‐mRuby2 to yield pcDNA3.1‐miRFP670nano3‐mScarlet3. For pcDNA3.1‐miRFP670nano3‐mRuby3, the mRuby3 open reading frame was ordered as a gene block and inserted into pcDNA3.1‐miRFP670nano3‐mRuby2 by digesting both with XhoI/BamHI followed by ligation.

pENTR221‐ShadowG and ‐Aquamarine were generated through first PCR amplification of the ShadowG and Aquamarine open reading frames from pCMV Aquamarine‐ShadowG tandem, prior to BP cloning into pDONR221 (ThermoFisher, 12536‐017) using BP clonase II (ThermoFisher, 11789), as per the manufacturer's instructions. Similarly, pENTR221‐mTurquoiseGL was generated by PCR amplification of mTurquoiseGL from 3568NES Eevee‐S6K and a BP reaction with pDONR221. All PCR reactions were performed using a high‐fidelity DNA polymerase (Phusion II HS, F549S, ThermoFisher) with the primers listed in Table S1. To generate the red‐shifted BioSrc (pENTR2b‐BioSrcFR), the pENTR2b‐BioSrcT plasmid was used to insert two gene blocks, the first miRFP670nano3 using HindIII/BspEI, then mRuby2 using BspEI/XbaI. For pENTR221‐miRFP670nano3 and ‐Eevee‐iAkt‐FR, miRFP670nano3 and the red‐shifted Eevee‐iAkt‐FR were ordered as gene blocks with flanking attB1/attB2 sites prior to a BP reaction with pDONR221. pENTR2b‐Eevee‐S6K was generated by restriction subcloning of 3568NES Eevee‐S6K with EcoRI/XhoI and ligation into a similarly digested pENTR2b. The pENTR221‐Eevee‐ROCK(WT, SSAA and SSDD) were generated through digestion of pENTR2b‐Eevee‐S6K with BspEI/NotI and ligation with annealed substrate fragments corresponding to the phosphorylation site in MLC2/*MYL2* (aa12‐26 GANSNVFSMFEQTQI; Table S1).

To generate final expression vectors, the pENTR221 or pENTR2b shuttle vectors were reacted with either pEF.DEST51, pPB.DEST or pLenti6.3/TO/V5‐DEST in an LR reaction using LR clonase II (ThermoFisher, 11791), as per the manufacturer's instructions. Constructs were all validated by analytical digests and sequencing, and are listed in Table S2. Gene blocks were synthesized by Integrated DNA technologies. Fast Digest restriction enzymes were purchased from ThermoFisher.

### 4.10 Stable Cell Line Generation Using PiggyBac Transposon Vectors

MM231 cells were co‐transfected with pCMV‐hyPBase and either pPB.DEST‐Eevee‐ROCK(WT), ‐Eevee‐ROCK(SSAA), ‐Eevee‐ROCK(SSDD), ‐Eevee‐BioSrcT, ‐Eevee‐Akt‐mT2, ‐Eevee‐iAkt‐mT2 or ‐Eevee‐S6K in a 2:1 ratio. Cells were then continuously cultured for 14 days prior to isolation by fluorescence activated cell sorting in a narrow fluorescence range.

### 4.11 Statistical Analysis

All statistical comparisons were made using Prism 9 (GraphPad software). All experiments were repeated at least three times independently. The choice to use parametric or nonparametric tests was made after first testing for normality using a D'Agostino‐Pearson omnibus test. Boxplots represent median and interquartile range, where the whiskers extend to min and max values. Robust z‐scores were calculated using the stats package (v3.6.2) in RStudio (v2022.12.10). Outliers were removed using ROUT (Q = 0.1%) in Prism 9 (GraphPad Software).

### 4.12 Western Blotting

Cell lysates were prepared in 1x RIPA lysis buffer (Merck, 20–188) with protease inhibitor cocktail (cOmpleteTM Mini, EDTA‐free, Roche) and Pierce Phosphatase Inhibitor Mini Tablets (A32957, Life Technologies). Separation was performed by gel electrophoresis (Mini‐PROTEAN TGX Precast Gels 4%–20%, Bio‐Rad, 4561096), prior to transfer onto a nitrocellulose membrane (Trans‐Blot Turbo Transfer System, Bio‐Rad) and blocking with StartingBlock Blocking Buffer (ThermoFisher, 37538). Primary antibodies in StartingBlock were incubated overnight at 4°C; Phospho‐Akt (Ser473) (D9E; Cell Signalling, 4060), Phospho‐Myosin Light Chain 2 (Ser19) (Cell Signalling, 3671), Phospho‐S6 Ribosomal Protein (Ser235/236) (D57.2.2E; Cell Signalling, 4858), RFP (Chromotek, 6G6), GAPDH (Hytest, 5G4‐6C5cc) or GFP (ThermoFisher, A11122). Membranes were washed between primary and secondary antibody treatments with TBST. HRP‐conjugated secondary antibodies (ThermoFisher, 1:5,000 diluted in TBST) were incubated for at least 1 h at room temperature, prior to detection on a GelDoc (Bio‐Rad). Quenching of HRP was performed with 30% hydrogen peroxide (Merck, 1072090500) between detections of secondary antibodies from different species. Densitometry analysis was performed in FIJI (NIH) by normalizing the signal to GAPDH, which was used as a loading control.

## Author Contributions

**Hasan Yassine**: investigation, methodology. **Van Thi‐Hong Tran**: investigation, methodology, formal analysis, writing – review and editing. **Vesa P. Hytönen**: writing – review and editing, supervision, funding acquisition. **Frans Ek**: investigation, formal analysis, methodology. **Rajalaxmi Behera**: methodology, investigation. **James R.W. Conway**: writing – review and editing, Writing – original draft, conceptualization, supervision, funding acquisition.

## Funding

This work was supported by a Research Council of Finland Academy Research Fellowship [360775], Research Council of Finland grant [363941], and the Cancer Foundation Finland (to J.R.W.C. and V.P.H.).

## Conflicts of Interest

The authors declare no conflicts of interest.

## Supporting information




**Supporting File**: smtd70828‐sup‐0001‐SuppMat.pdf.

**Supporting File**: smtd70828‐sup‐0002‐MovieS1.gif.

**Supporting File**: smtd70828‐sup‐0003‐MovieS2.gif.

**Supporting File**: smtd70828‐sup‐0004‐MovieS3.gif.

**Supporting File**: smtd70828‐sup‐0005‐MovieS4.gif.

**Supporting File**: smtd70828‐sup‐0006‐MovieS5.gif.

**Supporting File**: smtd70828‐sup‐0007‐MovieS6.gif.

## Data Availability

Computational modelling data is available upon request. All other study data are included in the article and supporting information.
