# Supplementary material for: Red‐to‐Near‐Infrared Fluorescence Resonance Energy Transfer Reporters for Dual Imaging Applications
Source: Small Methods. 2026 Jul 12;10(15):e70828. doi: 10.1002/smtd.70828 (PMC13450053; doi:10.1002/smtd.70828)
Supplement: Supplementary file 1 [file SMTD-10-e70828-s006.pdf]

Supporting Information

Red-to-near-infrared fluorescence resonance energy transfer reporters for dual imaging applications

Van Thi-Hong Tran[1], Frans Ek[2], Hasan Yassine[1], Rajalaxmi Behera[1,3], Vesa P. Hytönen[2,4] and James R.W. Conway[1,]*

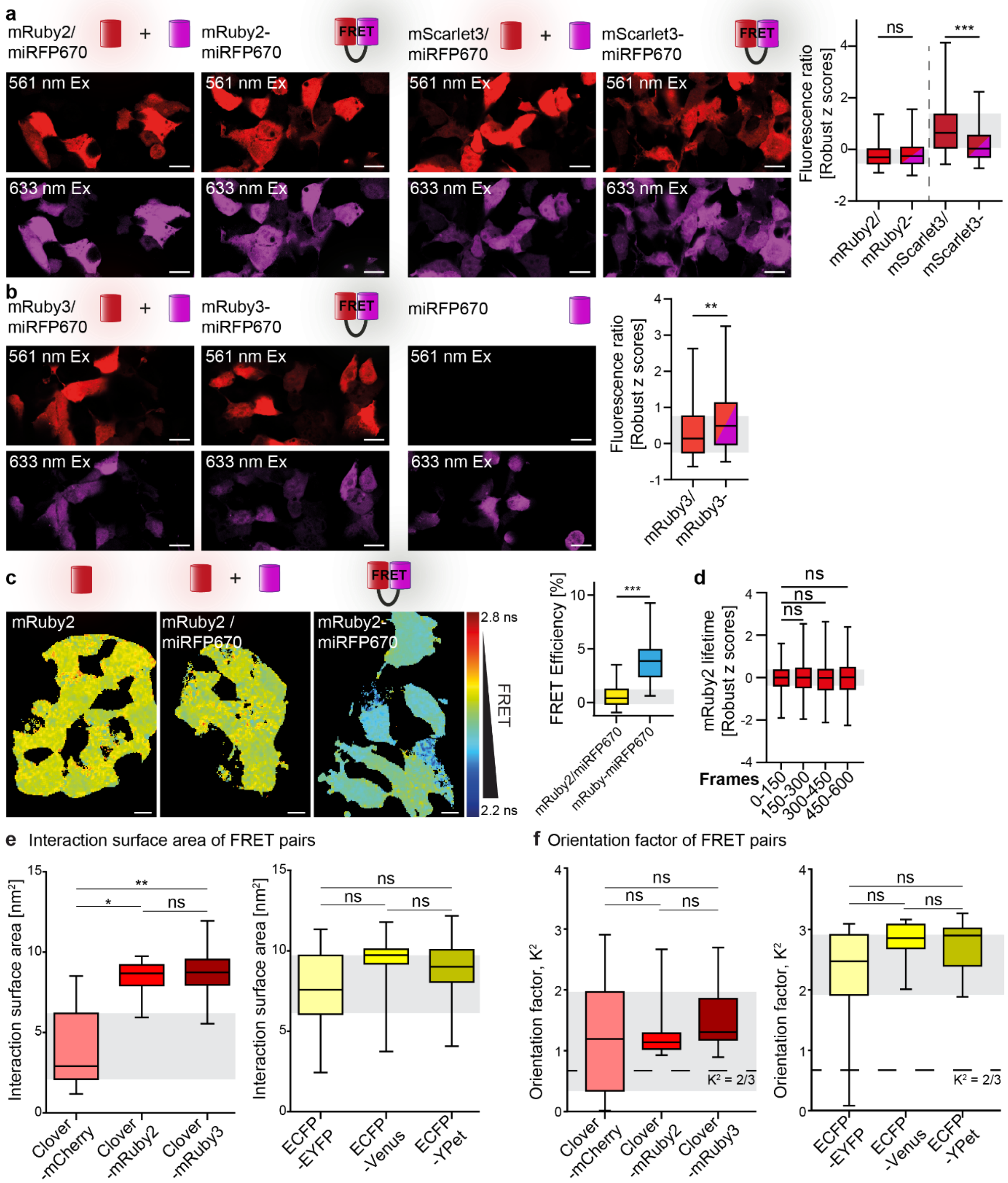

Figure S1. The red-to-NIR FRET pair mRuby2/miRFP670. (a) Representative images (left) and quantification (right) of mRuby2 with miRFP670 or mScarlet3 with miRFP670, demonstrating no loss of fluorescence of the proteins when tethered together (n=4 biological replicates, 27-63 cells/condition/replicate; p-values from a Kruskal-Wallis test with a Dunn's correction for multiple comparisons; ns, not significant; ***$p<0.001$; scale bars, 20 µm). (b) Representative images (left) and quantification (right) of mRuby3 with miRFP670, or miRFP670 alone, again demonstrating no loss of fluorescence of the proteins when tethered together and no detectable emission of miRFP670 in the 561 nm excitation channel (n=4 biological replicates, 12-45 cells/condition/replicate; p-values from a Mann-Whitney test; **$p<0.01$; scale bars, 20 µm). (c) Representative images (left) and FRET efficiencies (right) from HEK293FT cells transfected with mRuby2 (i.e. donor-only control) or either mRuby2 and miRFP670 or mRuby2-miRFP670 (n=3 biological replicates, 20-25 cells/condition/replicate;

p-values from a Mann-Whitney test; ***p<0.001; Scale bar, 20 μm). (d) Comparison of fluorescence lifetime after extended excitation across four consecutive 150-frame acquisition segments of the same imaging region (9-23 cells/replicate; p-values from a repeated measures one-way ANOVA with a Geisser-Greenhouse correction and a Holm-Šidák correction for multiple comparisons; ns, not significant). (e and f) Comparison of predicted (e) interaction surface areas and (f) orientation factors for the given pairs (25 simulations/pair; p-values from a Kruskal-Wallis test with a Dunn's correction for multiple comparisons; *p<0.05, **p<0.01; ns, not significant). Gray regions on boxplots indicate the interquartile range of the control condition. All boxplots include the median, interquartile range, and full data range (min-to-max whiskers).

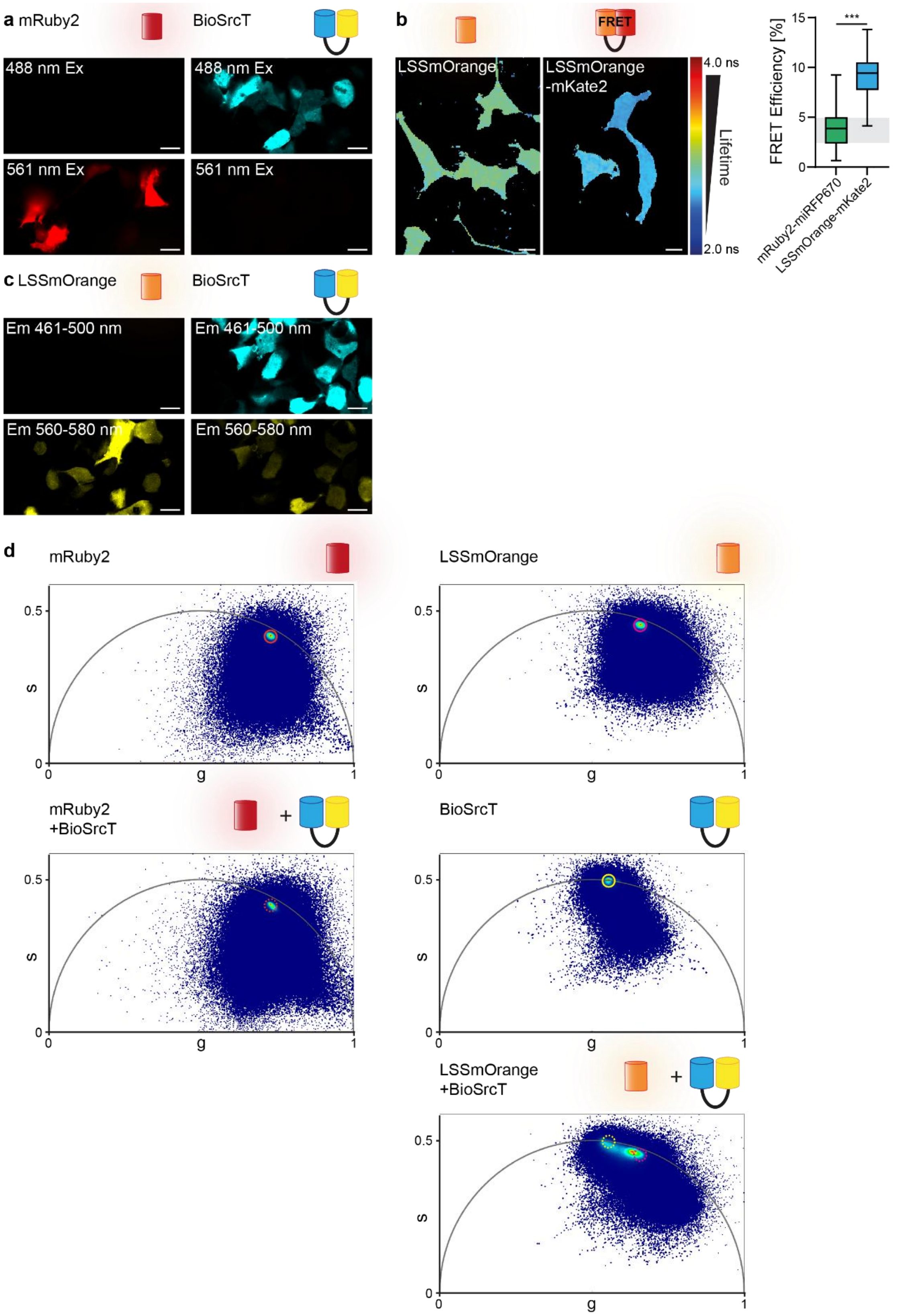

Figure S2. LSSmOrange shows FRET to mKate2, but bleed-through from co-expressed BioSrcT. (a) Representative images of HEK293FT cells transfected with either mRuby2 or BioSrcT and imaged with either 488 nm excitation and detecting fluorescence between 500-540 nm (488 nm Ex) or 561 nm excitation and detecting fluorescence between 570-610 nm (561 nm Ex; n=4 biological replicates, 5 fields of view per condition per replicate). Scale bar, 20 µm. (b) Representative images (left) and FRET efficiencies (right) from HEK293FT cells

transfected with LSSmOrange (i.e. donor-only control) or LSSmOrange-mKate2, compared to HEK293FT cells transfected with mRuby2 (i.e. donor-only control) or mRuby2-miRFP670 (n=3 biological replicates, 17-25 cells/condition/replicate; p-values from an unpaired Student's t test with a Welch's correction; ***p<0.001; Scale bar, 20 µm). The gray region on the boxplot indicates the interquartile range of the control condition. The boxplot includes the median, interquartile range, and full data range (min-to-max whiskers). (c) Representative images of HEK293FT cells transfected with either LSSmOrange or BioSrcT and imaged with 448 nm excitation and detecting fluorescence at either 461-500 nm or 560-580 nm (n=4 biological replicates, 5 fields of view per condition per replicate). Scale bar, 20 µm. (d) Representative phasor plots from data presented in **Figure 2A** (mRuby2+BioSrcT) and **Figure 2D** (LSSmOrange+BioSrcT), where each phasor plot is an average of 5 fields of view from a single representative biological replicate.

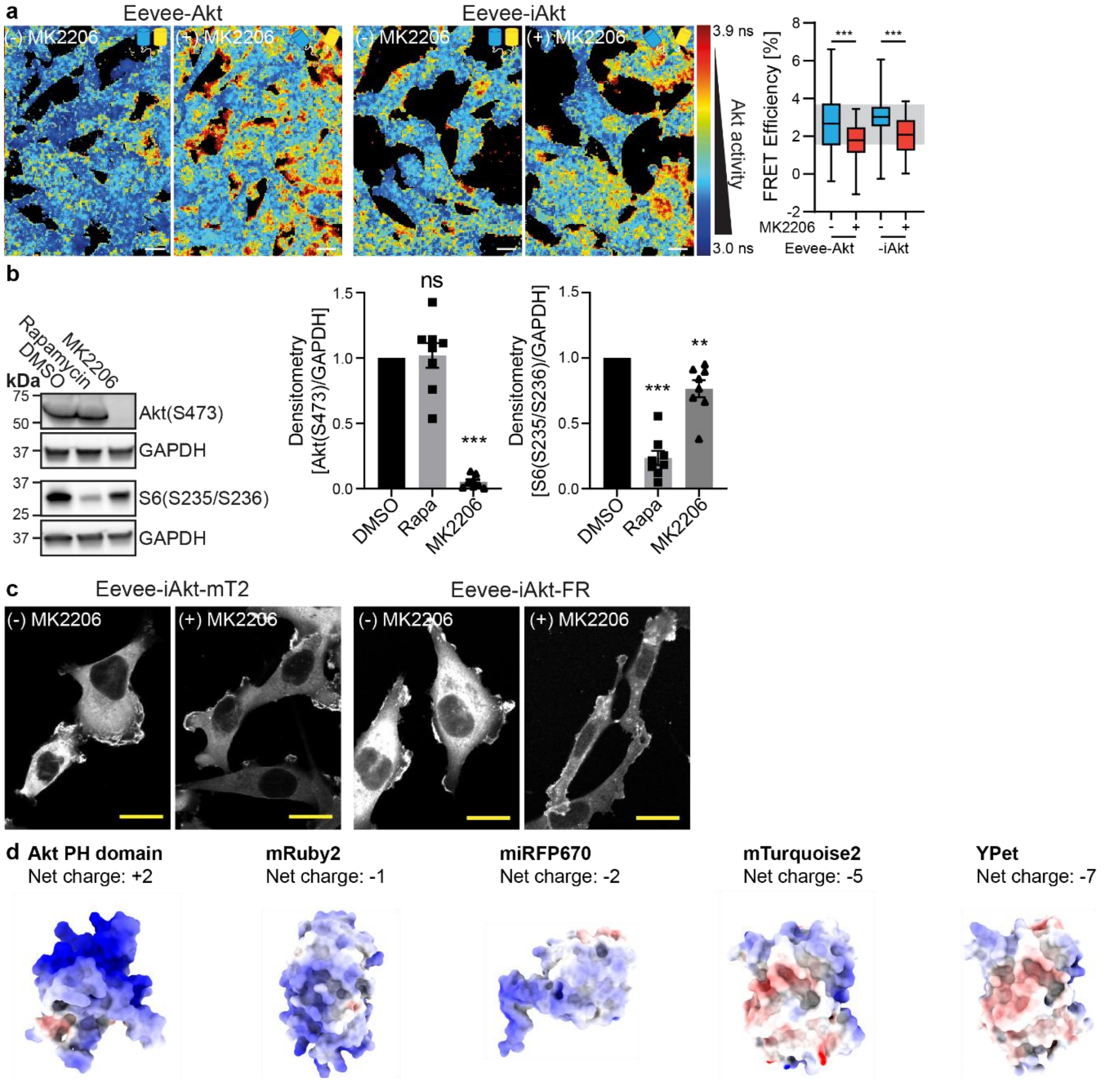

Figure S3. The GSK3β substrate demonstrates a small increase in dynamic range for Eevee-iAkt-mT2 over Eevee-Akt-mT2. (a) Representative images (left) and FRET efficiencies (right) from MM231 cells transfected with mTurquoise2 (i.e. donor-only control) or stably expressing either Eevee-Akt-mT2 or Eevee-iAkt-mT2 and treated with MK-2206 (500 nM) or DMSO control for 16 hours (22-25 cells/condition/replicate; n=3; p-values from a Kruskal-Wallis test with a Dunn's correction for multiple comparisons; ***p<0.001; Scale bars, 20 µm). The gray region on the boxplot indicates the interquartile range of the control condition. The boxplot includes the median, interquartile range, and full data range (min-to-max whiskers). (b) Representative western blots (left) and densitometry (right) of MM231 cells stably co-expressing Eevee-iAkt-FR and Eevee-S6K, treated with either rapamycin (100 nM) for 2 hours, or MK2206 (500 nM) or DMSO control for 16 hours (n=8; one-sample t-test; ns, not significant; **p<0.01; ***p<0.001). (c) Representative intensity images of MM231 cells expressing either Eevee-iAkt-mT2 or Eevee-iAkt-FR, and treated with MK-2206 (500 nM) or DMSO control for 16 hours. (d) The PH domain of Akt2, mRuby2, miRFP670, mTurquoise2 and YPet overlaid with their respective electrostatic potential calculated using the Adaptive Poisson-Boltzmann Solver (APBS) with a solute dielectric of 2 and a solvent dielectric of 78.54, then rendered using ChimeraX.

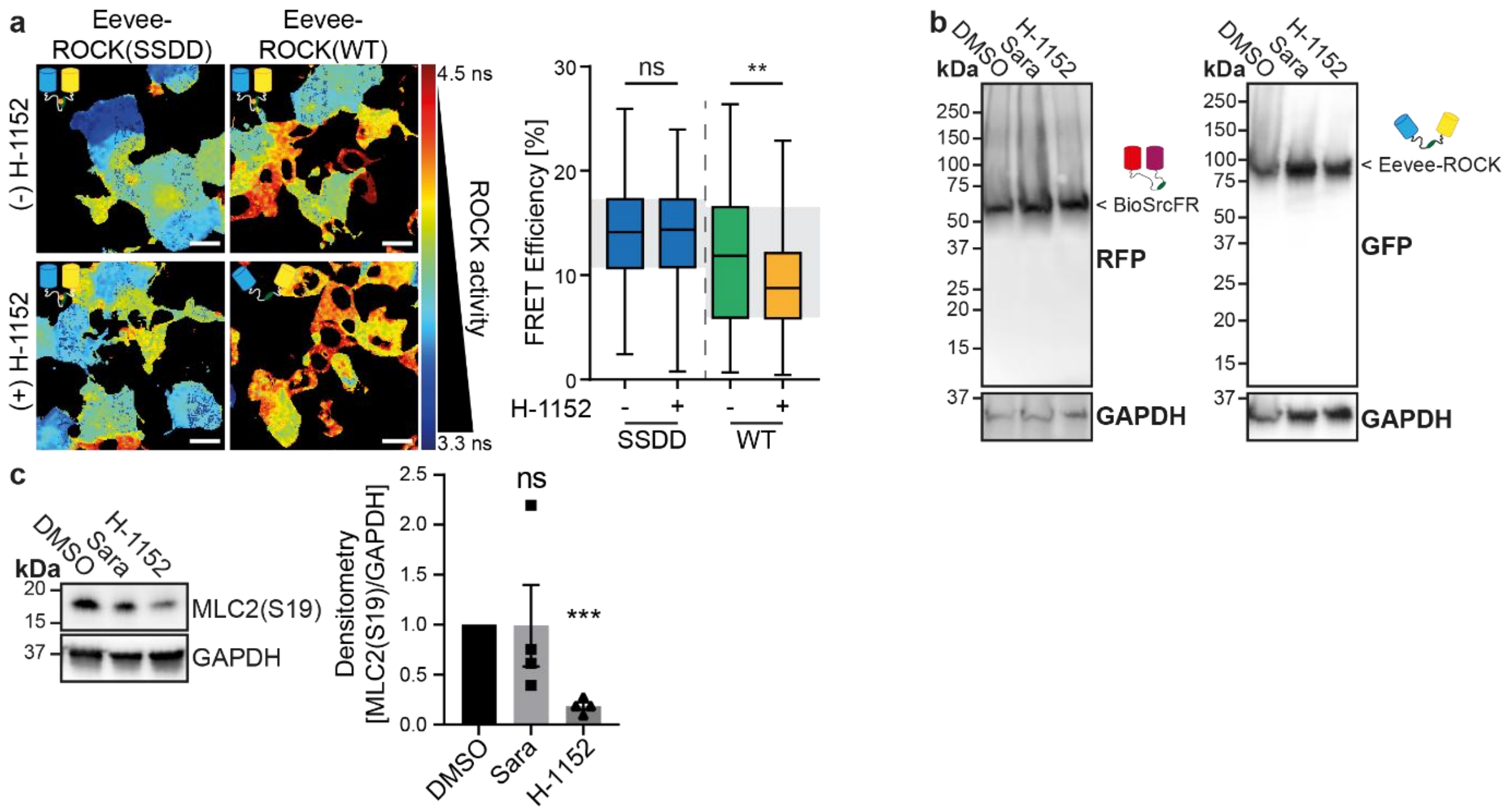

Figure S4. The Eevee-ROCK FRET reporter demonstrates phosphorylation-specific sensitivity to ROCK activity. (a) Representative images (left) and FRET efficiencies (right) from HEK293FT cells transfected with mTurquoiseGL (i.e. donor-only control) or Eevee-ROCK(WT/SSDD) and treated with H-1152 (5 µM) or DMSO for 2 hours (n=4 biological replicates, 17-31 cells/condition/replicate; p-values from a Kruskal-Wallis test with a Dunn's correction for multiple comparisons; ns, not significant; **p<0.01; scale bars, 20 µm). The gray region on the boxplot indicates the interquartile range of the control condition. The boxplot includes the median, interquartile range, and full data range (min-to-max whiskers). (b) Representative western blots of MM231 cells stably co-expressing Eevee-ROCK and BioSrcFR, treated with H-1152 (5 µM) for 2 hours, or Sara (1 µM) or DMSO for 24 hours (n=4). (c) Representative western blot (left) and densitometry (right) of MM231 cells stably co-expressing Eevee-ROCK and BioSrcFR, treated with H-1152 (5 µM) for 2 hours, or Sara (1 µM) or DMSO for 24 hours (n=4; one-sample t-test; ns, not significant; ***p<0.001).

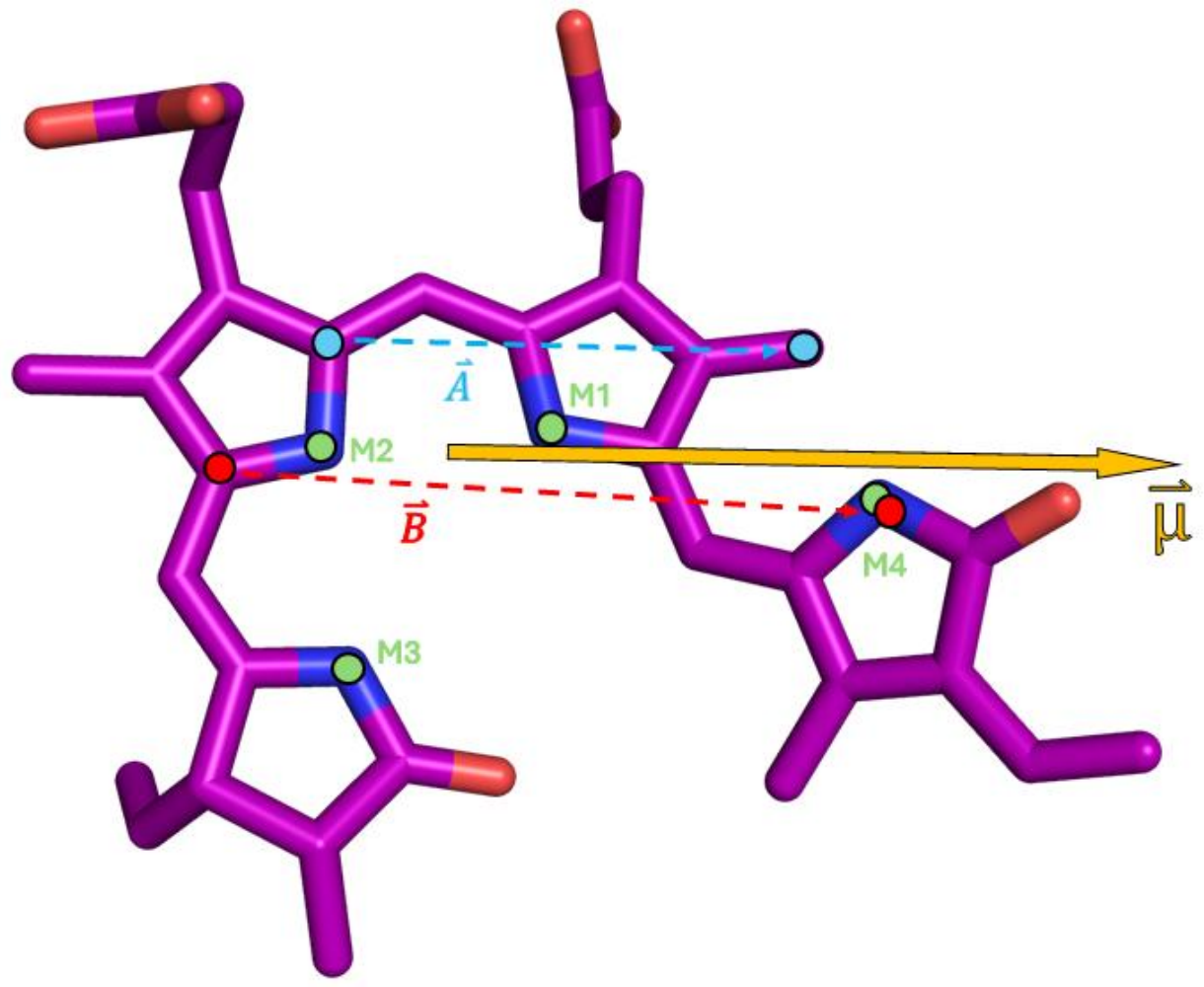

Figure S5. Atomic Selections used for calculating the orientation factor of miRFP670 chromophore (JRA). Transition dipole moment **μ** predicted with ORCA is presented as orange arrow. The transition dipole moment is approximated as in [48] by defining 2 vectors, **A** (blue dotted arrow) & **B** (red dotted arrow), from which the average vector is calculated. The center of mass for the whole chromophore is calculated as the center of mass for 4 atom selections: M1, M2, M3 and M4.

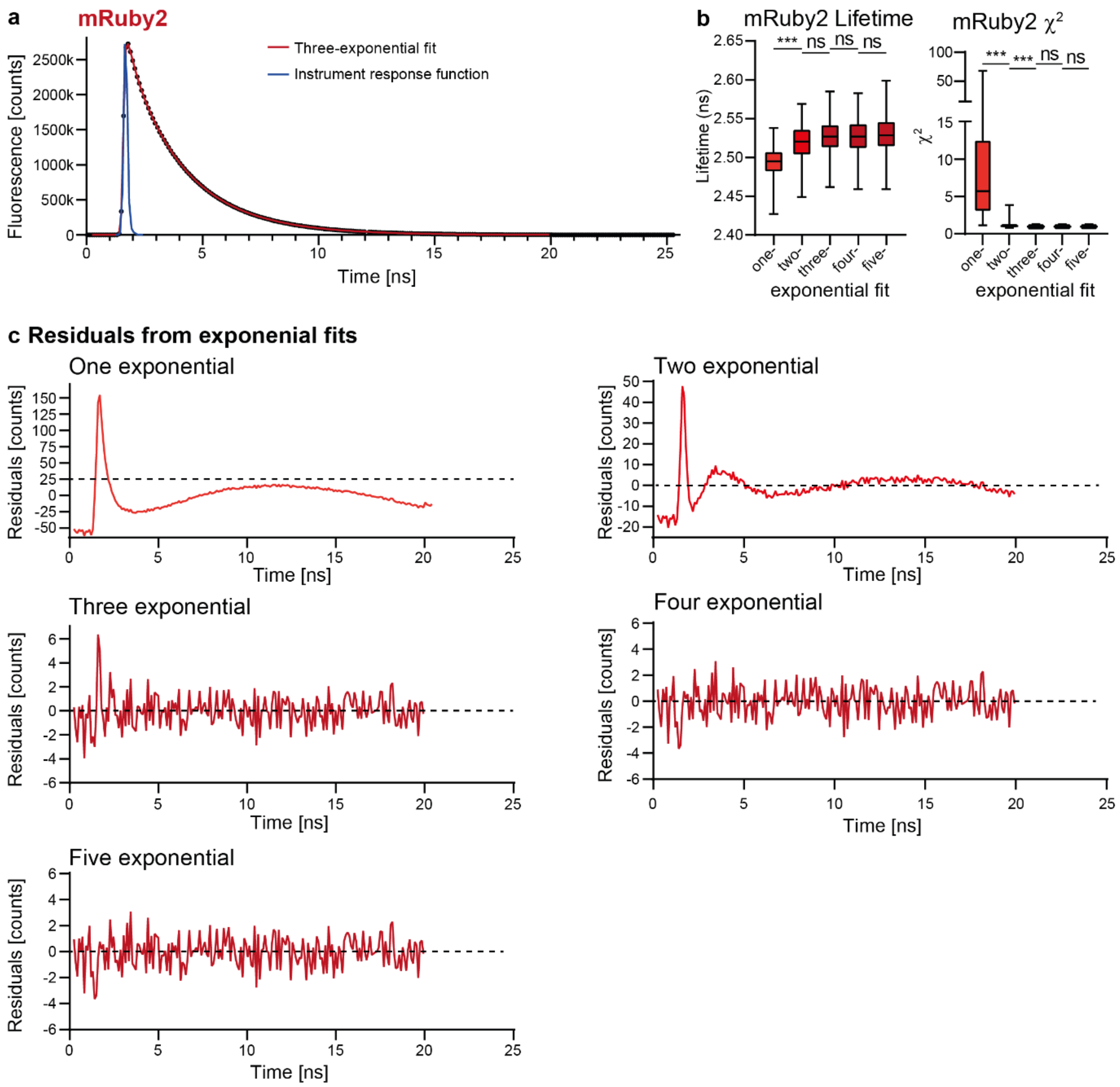

Figure S6. A three-exponential fitting approach provides the most robust readout of fluorescence lifetime for mRuby2 (n=4 biological replicates, 14-21 cells/condition/replicate; p-values from a one-way ANOVA test with a Holm-Šídák correction for multiple comparisons; ns, not significant; ***$p<0.001$). (a) Representative FLIM decay from mRuby2 transiently transfected into HEK293FT cells. (b) Lifetime measurements of mRuby2 using one-, two-, three-, four- and five-exponential fits (left) and their corresponding χ^2 values (right). All boxplots include the median, interquartile range, and full data range (min-to-max whiskers). (c) Representative residuals from the different fitting models applied to the data in (a).

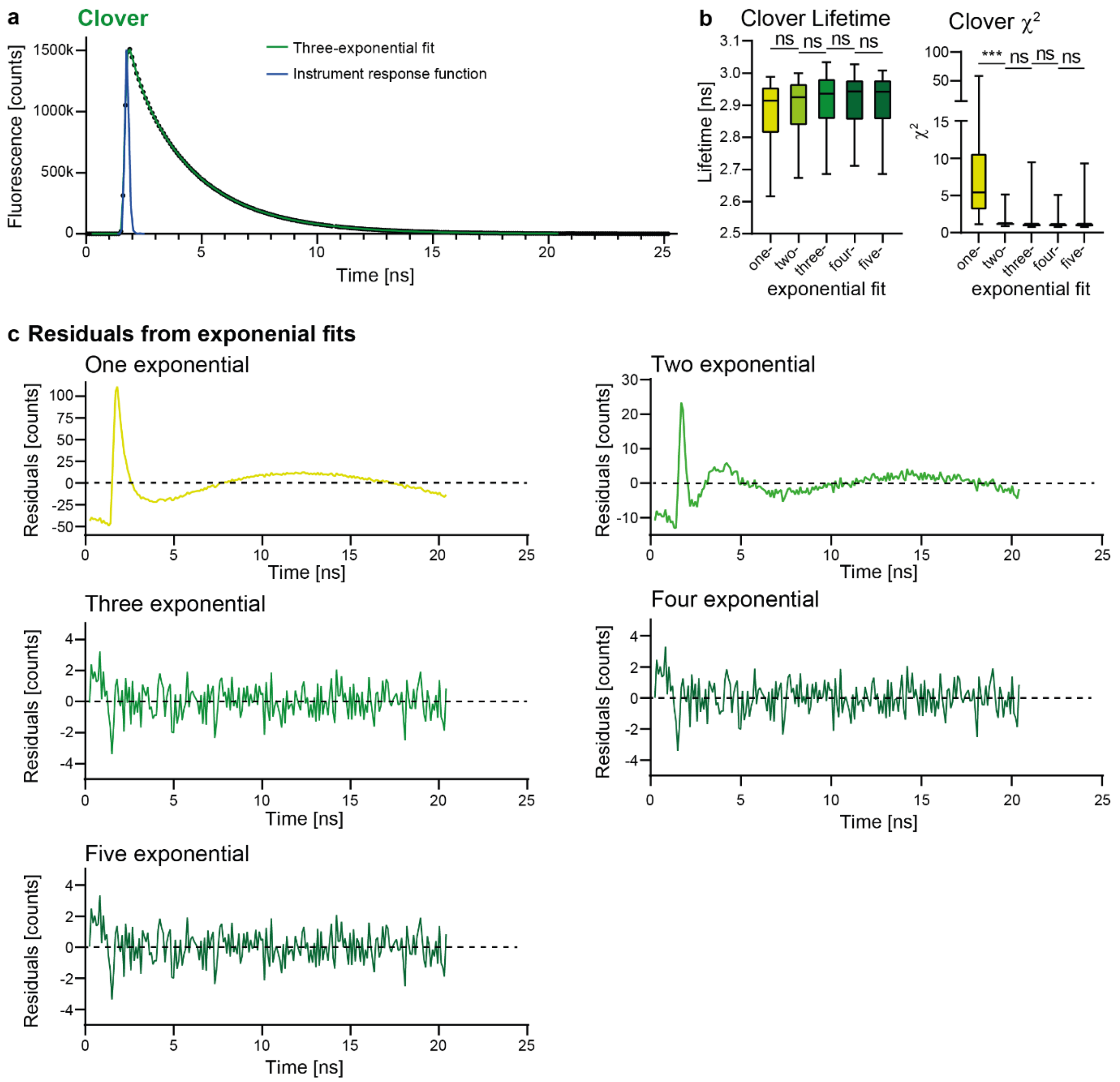

Figure S7. A three-exponential fitting approach provides the most robust readout of fluorescence lifetime for Clover (n=4 biological replicates, 13-20 cells/condition/replicate; p-values from a Kruskal-Wallis test with a Dunn's correction for multiple comparisons; ns, not significant; ***$p<0.001$). (a) Representative FLIM decay from Clover transiently transfected into HEK293FT cells. (b) Lifetime measurements of Clover using one-, two-, three-, four- and five-exponential fits (left) and their corresponding χ^2 values (right). All boxplots include the median, interquartile range, and full data range (min-to-max whiskers). (c) Representative residuals from the different fitting models applied to the data in (a).

Table S1. Oligonucleotides used for molecular cloning

Oligonucleotide	Sequence (5' to 3')
MYL2_top	ccggaggcgccaactccaacgtgttctccatgttcgaacagacccaaatcggc
MYL2_bottom	ggccgccgatttgggtctgttcgaacatggagaacacgttggagttggcgcct
MYL2(SSAA)_top	ccggaggcgccaacgccaacgtgttcgccatgttcgaacagacccaaatcggc
MYL2(SSAA)_bottom	ggccgccgatttgggtctgttcgaacatggcgaacacgttggcgttggcgcct
MYL2(SSDD)_top	ccggaggcgccaacGACaacgtgttcGACatgttcgaacagacccaaatcggc
MYL2(SSDD)_bottom	ggccgccgatttgggtctgttcgaacatGTCgaacacgttGTCgttggcgcct
Aquamarine_attB1_F	TAGAACAAGTTTGTACAAAAAAGCAGGCTtaccggtcgcc accatg
Aquamarine_attB2_R	TAGGGACCACTTTGTACAAGAAAGCTGGGTAgagtccgg acttgtacagc
ShadowG_attB1_F	TAGAACAAGTTTGTACAAAAAAGCAGGCTCcccgggatc cggaatggtgagcaa
ShadowG_attB2_R	TAGGGACCACTTTGTACAAGAAAGCTGGGTAGcgcttgt acagctcgtccatgccgagag
mTurquoiseGL_attB1_F	TAGAACAAGTTTGTACAAAAAAGCAGGCTCaggccgcA TGGTGAGCAAGGG
mTurquoiseGL_attB2_R	TAGGGACCACTTTGTACAAGAAAGCTGGGTAGcgATC CAGAGTCAGGCGTTCCAGAGGAG
mScarlet3_BamHI_F	CTggggatccgatggatagcaccgaggcagtgatcaagg
mScarlet3_EcoRI_R	tgcagaattcttaggagccaccggagccgcc

Table S2. Plasmid constructs used in this study

Plasmid	Addgene #	Reference
pDONR221	n/a	#12536017, ThermoFisher (Invitrogen)
pEF.DEST51	n/a	#12285011, ThermoFisher (Invitrogen)
pENTR2b	n/a	#A10463, ThermoFisher (Invitrogen)
pLenti6.3/TO/V5-DEST	n/a	ThermoFisher (Invitrogen)
pPB.DEST	n/a	[36]
pcDNA3-mRuby2	40260	[4]
pcDNA3-Clover	40259	[4]
pcDNA3.1-Clover-mRuby2	49089	A kind gift from Kurt Beam
pCMV-hyPBase	n/a	Wellcome Trust Sanger Institute [30]
pMDLg/pRRE	12251	A kind gift from Didier Trono (EPFL, Switzerland).
pRSV-Rev	12253	A kind gift from Didier Trono (EPFL, Switzerland).
pMD2.G	12259	A kind gift from Didier Trono (EPFL, Switzerland).
pENTR2b-mTurquoise2	191014	[65]
pPB.DEST-mTurquoise2	n/a	This work
pENTR221-ShadowG	252334	This work
pEF.DEST51-ShadowG	252335	This work
pENTR221-TurquoiseGL	252336	This work
pEF.DEST51-TurquoiseGL	252337	This work
pENTR221-miRFP670nano3	252338	This work
pEF.DEST51-miRFP670nano3	252339	This work
pENTR2b-EGFP	215537	[41]
pEF.DEST51-EGFP	252340	This work
pCMV Aquamarine -ShadowG tandem	157774	[66]
pENTR221-Aquamarine	252341	This work
pEF.DEST51-Aquamarine	252342	This work
pLSSmOrange-C1	37131	[27]
LSSmOrange2-mKate2	99868	[28]
mRuby3-C1	127808	[67]
pcDNA3.1-miRFP670nano3-mRuby2	252343	This work
pcDNA3.1-miRFP670nano3-mScarlet3	252344	This work
pmScarlet3-C1	189753	[68]
pcDNA3-miRFP670nano3-mRuby3	252345	This work
pPB.DEST-Eevee-Akt-mT2	n/a	[36]
pENTR221-Eevee-iAkt-mT2	252346	This work
pPB.DEST-Eevee-iAkt-mT2	252347	This work
pENTR221-Eevee-iAkt-FR	252348	This work
pLenti6.3-Eevee-iAkt-FR	252349	This work
3568NES Eevee-S6K	n/a	[34]

pENTR2b-Eevee-S6K	252350	This work
pPB.DEST-Eevee-S6K	252351	This work
pENTR2b-BioSrcT	n/a	[40]
pPB.DEST-BioSrcT	n/a	[40]
pENTR2b-BioSrcFR	252352	This work
pLenti6.3-BioSrcFR	252353	This work
pENTR2b-Eevee-ROCK(WT)	252354	This work
pPB.DEST-Eevee-ROCK(WT)	252355	This work
pENTR2b-Eevee-ROCK(SSAA)	252356	This work
pPB.DEST-Eevee-ROCK(SSAA)	252357	This work
pENTR2b-Eevee-ROCK(SSDD)	252358	This work
pPB.DEST-Eevee-ROCK(SSDD)	252359	This work

Additional references for supporting information

65. J. R. W. Conway, D. D. Dinc, G. Follain, O. Paavolainen, J. Kaivola, P. Bostrom, P. Hartiala, E. Peuhu, J. Ivaska, *Sci Adv* 2023**, 9** (28), eadg1840.

66. G. Bertolin, A. L. Bulteau, M. C. Alves-Guerra, A. Burel, M. T. Lavault, O. Gavard, S. Le Bras, J. P. Gagne, G. G. Poirier, R. Le Borgne, C. Prigent, M. Tramier, *Elife* 2018**, 7**.

67. V. Dunsing, M. Luckner, B. Zuhlke, R. A. Petazzi, A. Herrmann, S. Chiantia, *Sci Rep* 2018**, 8** (1), 10634.

68. T. W. J. Gadella, Jr., L. van Weeren, J. Stouthamer, M. A. Hink, A. H. G. Wolters, B. N. G. Giepmans, S. Aumonier, J. Dupuy, A. Royant, *Nature methods* 2023**, 20** (4), 541-545.

Legends for Movies S1-S6

Movie S1 (separate file). BioSrcFR FLIM in MM231s treated with DMSO. FLIM maps of the BioSrcFR FRET reporter in collagen-embedded MM231s stably both Eevee-ROCK and BioSrcFR, and treated with DMSO for 24 hours (scale bar, 20 µm).

Movie S2 (separate file). Eevee-ROCK FLIM in MM231s treated with DMSO. FLIM maps of the Eevee-ROCK FRET reporter in collagen-embedded MM231s stably both Eevee-ROCK and BioSrcFR, and treated with DMSO for 24 hours (scale bar, 20 µm).

Movie S3 (separate file). BioSrcFR FLIM in MM231s treated with H-1152. FLIM maps of the BioSrcFR FRET reporter in collagen-embedded MM231s stably both Eevee-ROCK and BioSrcFR, and treated with H-1152 for 2 hours (scale bar, 20 µm).

Movie S4 (separate file). Eevee-ROCK FLIM in MM231s treated with H-1152. FLIM maps of the Eevee-ROCK FRET reporter in collagen-embedded MM231s stably both Eevee-ROCK and BioSrcFR, and treated with H-1152 for 2 hours (scale bar, 20 µm).

Movie S5 (separate file). BioSrcFR FLIM in MM231s treated with Saracatenib. FLIM maps of the BioSrcFR FRET reporter in collagen-embedded MM231s stably both Eevee-ROCK and BioSrcFR, and treated with Saracatenib for 24 hours (scale bar, 20 µm).

Movie S6 (separate file). Eevee-ROCK FLIM in MM231s treated with Saracatenib. FLIM maps of the Eevee-ROCK FRET reporter in collagen-embedded MM231s stably both Eevee-ROCK and BioSrcFR, and treated with Saracatenib for 24 hours (scale bar, 20 µm).
